# Clinical outcomes in patients with relapsed/refractory *FLT3*-mutated acute myeloid leukemia treated with gilteritinib who received prior midostaurin or sorafenib

**DOI:** 10.1038/s41408-022-00677-7

**Published:** 2022-05-30

**Authors:** Alexander E. Perl, Naoko Hosono, Pau Montesinos, Nikolai Podoltsev, Giovanni Martinelli, Nicki Panoskaltsis, Christian Recher, Catherine C. Smith, Mark J. Levis, Stephen Strickland, Christoph Röllig, Marco Groß-Langenhoff, Wen-Chien Chou, Je-Hwan Lee, Hisayuki Yokoyama, Nahla Hasabou, Qiaoyang Lu, Ramon V. Tiu, Jessica K. Altman

**Affiliations:** 1 Abramson Cancer Center, University of Pennsylvania, Philadelphia, PA USA; 2 University of Fukui, Fukui, Japan; 3 Hospital Universitario y Politécnico La Fe, Valencia, Spain; 4 Yale School of Medicine, New Haven, CT USA; 5 Istituto Romagnolo per lo Studio dei Tumori “Dino Amadori” IRST S. r. l, Meldola, Italy; 6 Winship Cancer Institute, Emory University School of Medicine, Atlanta, GA USA; 7 Cancer Research Center of Toulouse, Toulouse, France; 8 University of California-San Francisco, San Francisco, CA USA; 9 The Sidney Kimmel Comprehensive Cancer Center, Johns Hopkins University, Baltimore, MD USA; 10 Vanderbilt Ingram Cancer Center, Nashville, TN USA; 11 Universitätsklinikum Carl Gustav Carus, Dresden, Germany; 12 Astellas Pharma GmbH, Munich, Germany; 13 National Taiwan University Hospital, Taipei, Taiwan; 14 Asan Medical Center, University of Ulsan College of Medicine, Seoul, Korea; 15 Sendai Medical Center, National Hospital Organization, Sendai, Japan; 16 Astellas Pharma US, Northbrook, IL USA; 17 Robert H. Lurie Comprehensive Cancer Center, Northwestern University Feinberg School of Medicine, Chicago, IL USA; 18 Present Address: Tohoku University, National Hospital Organization, Sendai, Japan

**Keywords:** Diseases, Medical research

## Abstract

The *fms*-like tyrosine kinase 3 (FLT3) inhibitor gilteritinib is indicated for relapsed or refractory (R/R) *FLT3*-mutated acute myeloid leukemia (AML), based on its observed superior response and survival outcomes compared with salvage chemotherapy (SC). Frontline use of FLT3 tyrosine kinase inhibitors (TKIs) midostaurin and sorafenib may contribute to cross-resistance to single-agent gilteritinib in the R/R AML setting but has not been well characterized. To clarify the potential clinical impact of prior TKI use, we retrospectively compared clinical outcomes in patients with R/R *FLT3-*mutated AML in the CHRYSALIS and ADMIRAL trials who received prior midostaurin or sorafenib against those without prior FLT3 TKI exposure. Similarly high rates of composite complete remission (CRc) were observed in patients who received a FLT3 TKI before gilteritinib (CHRYSALIS, 42%; ADMIRAL, 52%) and those without prior FLT3 TKI therapy (CHRYSALIS, 43%; ADMIRAL, 55%). Among patients who received a prior FLT3 TKI in ADMIRAL, a higher CRc rate (52%) and trend toward longer median overall survival was observed in the gilteritinib arm versus the SC arm (CRc = 20%; overall survival, 5.1 months; HR = 0.602; 95% CI: 0.299, 1.210). Remission duration was shorter with prior FLT3 TKI exposure. These findings support gilteritinib for *FLT3*-mutated R/R AML after prior sorafenib or midostaurin.

## Introduction

Tyrosine kinase inhibitors (TKIs) that target the *fms*-like tyrosine kinase 3 (FLT3) receptor have demonstrated activity in patients with acute myeloid leukemia (AML) harboring activating mutations in *FLT3* [1], namely, *FLT3* internal tandem duplications (*FLT3*-ITD) in the juxtamembrane domain and *FLT3* tyrosine kinase domain (*FLT3*-TKD) point mutations in the activation loop [2]. The effect of activating *FLT3* mutations on clinical outcomes varies based upon the presence of certain recurrent chromosomal translocations, co-mutations such as *NPM1*, *FLT3* mutation type, and *FLT3*-ITD to wild-type *FLT3* allelic ratio [3, 4]. Patients with *FLT3*-ITD mutations—particularly those with a high *FLT3*-ITD mutant to wild-type allelic ratio at initial diagnosis—have short remission duration and poor survival [4]. In contrast, *FLT3*-TKD mutations may share aggressive clinical features with *FLT3*-ITD mutations, but have inconsistent prognostic effects [5]. Although prognostically less important, secondary *FLT3*-TKD mutations can confer resistance to certain *FLT3* TKIs [6]. Notably, TKIs vary in their selectivity for the FLT3 receptor [7] and, to a certain extent, *FLT3*-TKD point mutations vary in their sensitivity to TKIs [6, 8]. Additionally, therapy with FLT3 inhibitors in patients with relapsed or refractory (R/R) AML who harbor *FLT3*-ITD mutations has been shown to promote drug-resistant clonal populations that contain secondary, on-target mutations in *FLT3* that confer resistance to multiple TKIs [9, 10]. Because R/R AML is demonstrably polyclonal in nature, FLT3 TKIs may elicit clonal pressure to select for drug-resistant tumor cell populations with additional mutations that promote leukemic growth independent of the activation state of the FLT3 kinase [11–13], as well as for clones that lack *FLT3* mutations entirely [11, 12].

First-generation FLT3 TKIs, midostaurin and sorafenib, display relatively limited selectivity for FLT3 and have relatively low potency in human plasma, which is thought to underlie their modest antileukemic efficacy when used as single agents in patients with R/R *FLT3*-mutated AML [14, 15]. However, the likelihood of response is improved by midostaurin and relapse rates decrease when either agent is combined with frontline 7 + 3 induction therapy in patients with newly diagnosed *FLT3*-mutated AML [16, 17]. Midostaurin in combination with chemotherapy was approved for this indication based on findings from the phase 3 RATIFY trial based upon an improvement in overall survival (OS) compared with placebo [17, 18]. Sorafenib is not approved for AML but is commonly used as maintenance therapy following allogeneic transplant, as supported by two randomized studies that show reductions in relapse rates and favorable effects on posttransplant relapse-free survival [19, 20]. Despite the observed benefit with first-generation FLT3 TKIs in the frontline setting, relapse is still common, especially in patients who are unable to undergo allogeneic transplantation [21].

The second-generation FLT3 TKIs, gilteritinib and quizartinib, have greater selectivity for the FLT3 receptor and, when administered continuously, appear to show fewer toxicities related to off-target effects compared with first-generation FLT3 TKIs [22]. Both agents have clinical activity that paired with demonstrated survival benefits compared to standard salvage chemotherapy (SC) when administered as single agents in patients with *FLT3*-mutated R/R AML [23–25]. However, while quizartinib has demonstrated activity against *FLT3*-ITD mutations, it is largely ineffective against *FLT3*-TKD mutations, which can emerge over time as a resistance mechanism [6, 10]. In contrast, gilteritinib has demonstrated activity against both *FLT3*-ITD and *FLT3*-TKD mutations [26, 27]. Despite this, secondary resistance to gilteritinib can develop from either on-target *FLT3* mutations in a gatekeeper *FLT*3 residue (F691L), as well as off-target mechanisms, such as emergence of *NRAS* or related mutations that activate mitogen-activated protein kinase (MAPK) signaling downstream of FLT3 [12, 26]. Recently published evidence has demonstrated these and other mechanisms of resistance in a subset of patients with newly diagnosed *FLT3*-mutated AML who received midostaurin in combination with induction chemotherapy [11].

Gilteritinib was approved as single-agent therapy for patients with *FLT3*-mutated R/R AML based on findings from the phase 3 ADMIRAL trial (NCT02421939), which evaluated the safety and efficacy of a daily dose of 120-mg gilteritinib against SC in this patient population [24, 28]. The 120-mg/day dose (with the possibility of escalation up to 200 mg/day in case of no response) was identified as the recommended dose for the ADMIRAL study based on findings from the phase 1/2 dose-escalation/expansion CHRYSALIS trial (NCT02014558) of 20–450-mg gilteritinib in a *FLT3*-mutation–enriched R/R AML patient population [25]. In the ADMIRAL trial, patients assigned to 120-mg gilteritinib had significantly longer median OS than those assigned to SC (9.3 months vs 5.6 months, respectively; hazard ratio (HR) for death, 0.64; 95% confidence interval [CI]: 0.49, 0.83; *P* < 0.001); higher rates of complete remission (CR) with full or partial hematologic recovery were also observed in the gilteritinib arm (34.0% vs 15.3%, respectively) [24].

Because clonal evolution in AML has recently been shown to contribute to the development of resistance to initial FLT3 TKI therapy [11, 12] and there are limited data to guide clinical use of gilteritinib in an era where patients are commonly treated with frontline TKIs, we performed a retrospective analysis of the CHRYSALIS and ADMIRAL trials to evaluate response and survival in patients with *FLT3*-mutated, R/R AML who received or did not receive prior TKI therapy with midostaurin or sorafenib before treatment with gilteritinib.

## Material and methods

### CHRYSALIS and ADMIRAL study designs

CHRYSALIS was a multicenter phase 1/2 dose-escalation/expansion trial (start date: October 9, 2013; primary completion date: August 4, 2017) in which patients were enrolled across seven dose-escalation cohorts to receive 20-, 40-, 80-, 120-, 200-, 300-, and 450-mg doses of once-daily oral gilteritinib in 28-day cycles [25]. On the basis of emerging toxicity, pharmacokinetic and pharmacodynamic profile, and antileukemic response, the 120- and 200-mg dose-escalation cohorts were further expanded to include *FLT3*-mutated patients only [25]. A full description of the CHRYSALIS study design has been previously published by Perl and colleagues [25].

ADMIRAL was a global phase 3 trial (start date: October 20, 2015; primary completion date: September 17, 2021) of gilteritinib versus SC in patients with R/R *FLT3*-mutated AML [24]. Patients were randomized 2:1 to receive 120-mg gilteritinib or preselected high- or low-intensity SC [24]. Patients in the SC arm assigned to high-intensity SC received one to two cycles of treatment [24]. Treatment with gilteritinib or low-intensity chemotherapy was administered in 28-day cycles until disease progression or another discontinuation criterion was met [24]. Dose escalation to 200 mg/day was permitted for patients in the gilteritinib arm who did not have protocol-defined remission after the first treatment cycle. Complete details of the ADMIRAL study design and treatment are outlined in the primary publication [24]. The study protocols for the CHRYSALIS and ADMIRAL trials were approved by site-specific independent ethics committees or institutional review boards. All patients in the CHRYSALIS and ADMIRAL trials provided written informed consent at the time of enrollment.

### CHRYSALIS and ADMIRAL patient populations

Adult patients with R/R *FLT3*-mutated AML who received 120-mg or 200-mg gilteritinib in the phase 1/2 CHRYSALIS trial and those who received 120-mg gilteritinib or SC in the ADMIRAL trial were included in this analysis. The subgroup of R/R AML patients who received 120- or 200-mg gilteritinib in the CHRYSALIS trial had locally confirmed *FLT3* mutations and had received one or more lines of prior AML therapy [25]. Patients in the ADMIRAL trial had either relapsed after initial induction therapy or were refractory to initial induction therapy and were required to have central laboratory confirmed *FLT3*-ITD mutations or *FLT3*-TKD D835/I836 point mutations at study entry[24]; enrollment based on local *FLT3* mutation testing was permitted in cases of rapidly proliferative disease. Complete inclusion and exclusion criteria for patients in both trials are outlined in the respective primary publications [24, 25].

### Assessments

Treatment response was assessed using modified International Working Group criteria [29]. Complete definitions of treatment response parameters are presented in the Supplement (Table S1). The composite CR (CRc) rate was defined as the sum of patients who achieved CR, CR with incomplete hematologic recovery (CRi), and CR with incomplete platelet recovery (CRp). In the CHRYSALIS trial, *FLT3* mutation status was determined based on local testing. In the ADMIRAL trial, *FLT3* mutation status was assessed at enrollment (baseline) by a central laboratory using a polymerase chain reaction–based assay (LeukoStrat CDx) according to published methods; *FLT3* mutation status based on local testing was permitted in cases of rapidly proliferative disease [24, 30].

### Statistical analyses

Descriptive statistics were used to assess continuous variables. Categorical data were reported as frequencies and percentages. The Kaplan-Meier method and the Greenwood formula were used to estimate OS. Hazard ratio and supporting CIs were used to determine differences in OS between groups. As the statistical analysis plan did not include provisions for multiplicity correction with respect to evaluation of secondary outcomes or subgroup analyses, these results were reported as point estimates with 95% CIs. Statistical analyses were performed with SAS v9.3 or higher software.

## Results

### Baseline characteristics

Overall, 33 of 145 (22.8%) patients received 120- or 200-mg gilteritinib in the CHRYSALIS trial and 33 of 247 (13.4%) patients who received 120-mg gilteritinib in the ADMIRAL trial had received prior TKI therapy (Table 1). In the SC arm of the ADMIRAL trial, 15 patients had received prior TKI therapy. All patients who received prior TKIs in the 120- or 200-mg gilteritinib dose groups of the CHRYSALIS trial had received sorafenib. In the gilteritinib arm of the ADMIRAL trial, 58% (*n* = 19/33) of prior TKI-treated patients had received sorafenib and 42% (*n* = 14/33) had received midostaurin. Among prior TKI-treated patients in the SC arm of the ADMIRAL trial, 60% (*n* = 9/15) had received midostaurin and 40% (*n* = 6/15) had received sorafenib. For prior TKI-treated patients in the CHRYSALIS trial, the median time since last TKI therapy was 33 days (interquartile range [IQR], 16–149) for all patients who received 120-mg or 200-mg gilteritinib. For prior TKI-treated patients in the gilteritinib arm of the ADMIRAL trial, the median time since last TKI therapy was 34 days (IQR, 11–92). Demographic and baseline disease characteristics in prior TKI and no prior TKI subgroups within the CHRYSALIS and ADMIRAL trials were similar (Table 1). Among patients who received 120- or 200-mg gilteritinib in the CHRYSALIS trial, most (69%; *n* = 100/145) had received two or more lines of prior AML therapy. Baseline co-mutations in *NPM1* occurred slightly more frequently among patients in the SC arm of the ADMIRAL trial who had received prior TKI therapy (64%) compared with corresponding patients in the gilteritinib arm (47%) and patients who had not received prior TKI therapy (gilteritinib, 48%; SC, 45%). Baseline co-mutations in *RAS/MAPK* pathway genes (ie, *BRAF*, *CBL*, *KRAS*, *NRAS*, or *PTPN11*) were observed in seven patients (15%) who had received prior TKI therapy (gilteritinib, *n* = 5; SC, *n* = 2) and in 18 patients (6%) without prior TKI exposure (gilteritinib, *n* = 13; SC, *n* = 5).

**Table 1:** Baseline and Prior Treatment Characteristics of Patients With R/R AML in the CHRYSALIS and ADMIRAL Trials.

Characteristic	CHRYSALIS 120-/200-mg Gilteritinib		ADMIRAL 120-mg Gilteritinib vs salvage chemotherapy			
	Prior TKI (*n* = 33)	No Prior TKI (*n* = 112)	Gilteritinib		Salvage chemotherapy	
			Prior TKI (*n* = 33)	No Prior TKI (*n* = 214)	Prior TKI (*n* = 15)	No Prior TKI (*n* = 109)
Median age, years (range)	56 (24–84)	61 (22–87)	55 (20–82)	62.5 (22–84)	64 (34–78)	61 (19–85)
Female, *n* (%)	18 (55)	59 (53)	14 (42)	117 (55)	9 (60)	61 (56)
ECOG performance status, *n* (%)						
0–1	22 (67)	87 (78)	25 (76)	181 (85)	14 (93)	91 (84)
≥2	11 (33)	25 (22)	8 (24)	33 (15)	1 (7)	18 (17)
*FLT3* mutation type, *n* (%)						
*FLT3*-ITD only	29 (88)	94 (84)	24 (73)	191 (89)	14 (93)	99 (91)
*FLT3*-TKD only	0	9 (8)	5 (15)	16 (8)	1 (7)	9 (8)
*FLT3*-ITD and -TKD	4 (12)	7 (6)	4 (12)	3 (1)	0	0
Other/unknown/missing	0	2 (2)	0	4 (2)	0	1 (0.9)
Cytogenetic risk status, *n* (%)						
Favorable	0	4 (4)	0	4 (2)	0	1 (0.9)
Intermediate	23 (70)	78 (70)	30 (91)	152 (71)	14 (93)	75 (69)
Unfavorable	4 (12)	14 (13)	3 (9)	23 (11)	1 (7)	10 (9)
Other/unknown/missing	6 (18)	16 (14)	0	35 (16)	0	23 (21)
Response to first-line therapy, *n* (%)						
Relapsed	22 (67)	74 (66)	19 (61)	130 (61)	12 (80)	64 (59)
Primary refractory	11 (33)	38 (34)	14 (42)	84 (39)	3 (20)	45 (41)
Prior lines of therapy, *n* (%)						
1	3 (9)	42 (38)	33 (100)	215 (100)	15 (100)	109 (100)
2	6 (18)	36 (32)	0	0	0	0
≥3	24 (73)	34 (30)	0	0	0	0
Prior TKI, *n* (%)						
Midostaurin	0	NA	14 (42)	NA	9 (60)	NA
Sorafenib	33 (100)		19 (58)		6 (40)	
Prior HSCT, *n* (%)						
Yes	14 (42)	34 (30)	10 (30)	38 (18)	4 (27)	22 (20)
No	19 (58)	78 (70)	23 (69)	176 (82)	11 (73)	87 (80)
On-study HSCT, *n* (%)						
Yes	6 (18)	24 (21)	5 (15)	59 (28)	0	19 (17)
No	27 (82)	88 (79)	29 (88)	155 (72)	15 (100)	90 (83)
Posttransplant gilteritinib maintenance therapy, *n* (%)						
Yes	0	12	4	36	NA	NA
No	6	12	1	23	NA	NA
Molecular Profile of Patients in the ADMIRAL Trial						
*FLT3*-ITD allelic ratio^a^, *n* (%)			*n* = 28	*n* = 194	*n* = 14	*n* = 99
High			14 (50)	95 (49)	6 (43)	54 (55)
Low			14 (50)	99 (51)	8 (57)	45 (45)
Co-mutations, *n* (%)			*n* = 32	*n* = 207	*n* = 14	*n* = 108
*NPM1*			15 (47)	100 (48)	9 (64)	49 (45)
*DNMT3A*			9 (28)	66 (32)	6 (43)	34 (31)
*DNMT3A* and *NPM1*			7 (22)	48 (23)	4 (29)	27 (25)
*WT1*			10 (31)	35 (17)	4 (29)	16 (15)
*IDH1 or IDH2*			4 (13)	34 (16)	4 (29)	14 (13)

^a^Measured as the ratio of *FLT3*-ITD to *FLT3* wild-type for all patients with a centrally confirmed *FLT3*-ITD mutation. A high *FLT3*-ITD allelic ratio was greater than or equal to the median value of 0.77 and a low *FLT3*-ITD allelic ratio was less than the median value of 0.77.

*AML* acute myeloid leukemia, *ECOG* Eastern Cooperative Oncology Group, *HSCT* hematopoietic stem cell transplantation, *IRT* interactive response technology, *ITD* internal tandem duplication, *NA* not applicable, *R/R* relapsed or refractory, *TKD* tyrosine kinase domain, *TKI* tyrosine kinase inhibitor.

### Survival outcomes

**Fig. 1:**
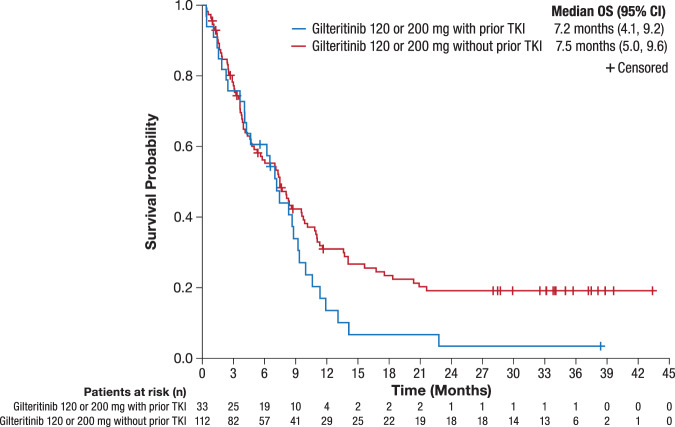
Overall survival by prior TKI status: CHRYSALIS trial. CI confidence interval, OS overall survival, R/R relapsed or refractory, TKI tyrosine kinase inhibitor.

Median OS was similar in prior TKI-treated and no prior TKI subgroups (7.2 months and 7.5 months, respectively) following treatment with 120- or 200-mg gilteritinib in the CHRYSALIS trial (Fig. 1). Median OS by *FLT3* mutation type is shown in Table S2.

**Fig. 2:**
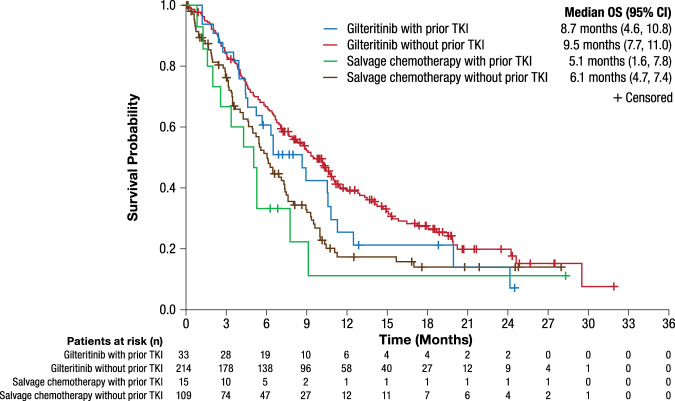
Overall survival by prior TKI status in patients with *FLT3*-mutated R/R AML: ADMIRAL trial. AML acute myeloid leukemia, CI confidence interval, OS overall survival, R/R relapsed or refractory, TKI tyrosine kinase inhibitor.

Among patients in the gilteritinib arm of the ADMIRAL trial, median OS duration was 9.5 months for those who did not receive prior TKIs compared with 8.7 months for those who received prior TKI therapy (Fig. 2). Among patients who received prior TKI therapy, a trend toward longer median overall survival (OS; 8.7 months) was observed in the gilteritinib arm than corresponding patients in the SC arm (median OS, 5.1 months; HR = 0.602; 95% CI: 0.299, 1.210). Among patients who did not receive prior TKI therapy, those in the gilteritinib arm had longer median OS (9.5 months) compared with those in the SC arm (6.1 months) (HR = 0.637; 95% CI: 0.482, 0.841). Median OS by *FLT3* mutation type in patients treated with gilteritinib in the ADMIRAL trial (Table S2) did not show any trend. Among patients who had primary refractory AML, median OS was similar among those who received prior TKI therapy (10.6 months) and those who did not (10.2 months) (Table S3). Among patients with relapsed AML, median OS was 6.5 months in those treated with prior TKIs and 8.9 months in those who did not receive prior TKI therapy.

**Fig. 3:**
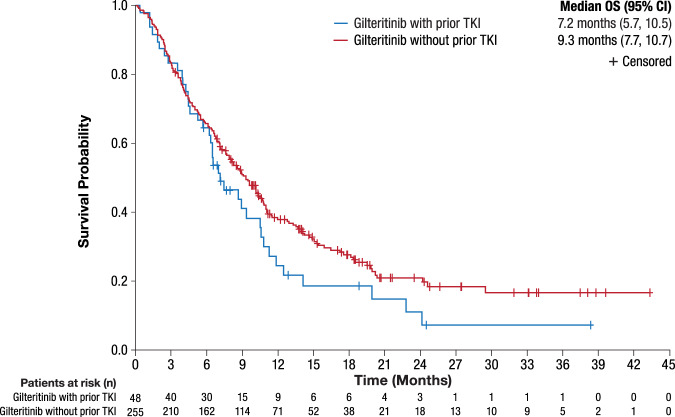
Overall survival by prior TKI status in patients with R/R AML treated with gilteritinib: CHRYSALIS and ADMIRAL trials combined (120 mg only). AML acute myeloid leukemia, CI confidence interval, OS overall survival, R/R relapsed or refractory, TKI tyrosine kinase inhibitor.

In the combined analysis of patients who received 120-mg gilteritinib in both trials, patients who did not receive prior TKI therapy had longer median OS duration than those who received prior TKI therapy (Fig. 3). Median EFS was similar for prior TKI and no prior TKI patients in the CHRYSALIS trial (3.6 months and 4.1 months, respectively). Likewise, in patients treated with 120-mg gilteritinib in the ADMIRAL trial, median EFS was the same (2.8 months) for both patients who received and did not receive prior TKI therapy.

### Response outcomes

Overall, more than 40% of patients who received 120- or 200-mg gilteritinib in the CHRYSALIS and ADMIRAL trial after treatment with prior TKIs achieved CRc. Rates of CRc among gilteritinib-treated patients were not markedly different between prior TKI and no prior TKI subgroups (Table 2). In the CHRYSALIS trial, the overall CRc rate (includes pre- and posttransplant CRc) was 42% after prior treatment with sorafenib, with all patients achieving CRc prior to transplantation. Median durations of CR and CRc were shorter in the prior TKI group (9.1 and 1.9 months, respectively) than in the no prior TKI group (15.8 and 6.3 months, respectively). The median duration of CR in patients previously treated with sorafenib was 9.1 months. The cumulative incidence of relapse at 12 months after achievement of CR in patients previously treated with sorafenib was 100%; the cumulative incidence of relapse at 12 months after achievement of CRc in these patients was 79.2%. In patients treated with prior sorafenib, most relapses occurred within the first 4 months after CRc; in patients not previously treated with sorafenib, most relapses occurred within the first 10 months after CRc (data not shown).

**Table 2:** Response outcomes in patients with R/R AML based on prior TKI therapy status in the CHRYSALIS and ADMIRAL trials.

Response parameter	CHRYSALIS 120-/200-mg Gilteritinib		ADMIRAL 120-mg Gilteritinib vs Salvage Chemotherapy			
	Prior TKI (*n* = 33)	No Prior TKI (*n* = 112)	Gilteritinib		Salvage Chemotherapy	
			Prior TKI (*n* = 33)	No Prior TKI (*n* = 214)	Prior TKI (*n* = 15)	No Prior TKI (*n* = 109)
Response rate^a^, *n* (%)						
CR	1 (3)	16 (14)	6 (18)	46 (21)	0	13 (12)
CRi	10 (30)	25 (22)	7 (21)	56 (26)	3 (20)	11 (10)
CRp	3 (9)	7 (6)	4 (12)	15 (7)	0	0
PR	2 (6)	9 (8)	5 (16)	28 (13)	1 (7)	4 (4)
NR	15 (45)	43 (38)	9 (27)	57 (26)	5 (33)	38 (35)
NE	2 (6)	12 (11)	2 (6)	12 (6)	6 (40)	43 (39)
**CRc**^**b**^	**14 (42)**	**48 (43)**	**17 (52)**	**117 (55)**	**3 (20)**	**24 (22)**
Median duration of CR, months (range)	9.1 (9.1 to 9.1)	15.8 (2.1 to 15.8)	8.9 (0.7+ to 15.7 + )	NE (0.6 to 23.1 + )	NA	1.8 (<0.1+ to 1.8)
Median duration of CRc, months (range)	1.9 (0.3 to 12.6)	6.3 (0.9 to 15.8)	3.7 (0.7 to 15.7 + )	4.8 (<0.1+ to 30.3 + )	NE (<0.1+ to 0.3 + )	NE (<0.1+ to 3.7 + )
**By TKI Agent**	**Sorafenib (*n*** = **33)**		**Midostaurin (*n*** = **14)**	**Sorafenib (*n*** = **19)**	**Midostaurin (*n*** = **9)**	**Sorafenib (*n*** = **6)**
Response rate^a^						
CR	1 (3)		3 (21)	3 (16)	0	0
CRi	10 (30)		4 (29)	3 (16)	3 (33)	0
CRp	3 (9)		1 (7)	3 (16)	0	0
PR	2 (6)		0	5 (26)	1 (11)	0
NR	15 (45)		5 (36)	4 (21)	3 (33)	2 (33)
NE	2 (6)		1 (7)	1 (5)	2 (22)	4 (67)
**CRc**^**b**^	14 (42)		8 (57)	9 (47)	3 (33)	0
Median duration of CR, months (range)	9.1 (9.1 to 9.1)		3.7 (0.7+ to 3.7)	12.9 (4.9 to 15.7 + )	NA	NA
Median duration of CRc, months (range)	1.9 (0.3 to 12.6)		3.0 (0.7 to 3.7)	4.6 (1.0+ to 15.7 + )	NE (0.1+ to 0.3 + )	NA

Bold font indicates aggregate responses.

^a^Includes pretransplant and posttransplant response. ^b^Defined as the sum of patients who achieved CR, CRi, and CRc.

*AML* acute myeloid leukemia, *CR* complete remission, *CRc* composite complete remission, *CRi* complete remission with incomplete hematologic recovery, *CRp* complete

remission with incomplete platelet recovery, *NA* not applicable, *NE* not evaluable, *NR* no response, *PR* partial remission, *R/R* relapsed or refractory, *TKI* tyrosine kinase inhibitor.

In the gilteritinib arm of the ADMIRAL trial, the CRc rate was 57% in patients treated with prior midostaurin, with 21% achieving CR, 29% achieving CRi, and 7% achieving CRp. In ADMIRAL patients who received gilteritinib after prior sorafenib, the overall CRc rate was 47% with equal proportions of patients achieving CR, CRi, and CRp (all 16%) (Table 2). Notably, overall CRc rates in the gilteritinib arm after prior midostaurin (57%) or sorafenib (47%) were higher than CRc rates in corresponding prior TKI-treated subgroups in the SC arm (33% and 0%, respectively). The pretransplant CRc rates in the gilteritinib arm after prior midostaurin or prior sorafenib were 50% and 42%, respectively. Rates of CRc in prior TKI-treated patients were 47% for patients in relapse at baseline and 57% in patients who were refractory at baseline (Table S3); similarly high rates of CRc were observed among patients without prior TKI exposure within relapsed (60%) and refractory (46%) subgroups. As observed in the CHRYSALIS trial, median durations of CR and CRc in the gilteritinib arm of the ADMIRAL trial were shorter in patients who had received prior TKIs than in patients who had not. Patients in the gilteritinib arm who were previously treated with sorafenib had a median duration of CR of 12.9 months and those who were previously treated with midostaurin had a median duration of CR of 3.7 months. In the gilteritinib arm, all relapses occurred within the first 4 months after CRc in patients treated with prior midostaurin; in patients treated with prior sorafenib, most relapses occurred within the first 5 months. Among patients in the gilteritinib arm who did not receive prior TKI therapy, most relapses occurred within the first 12 months after achievement of CRc (data not shown). The cumulative incidence of relapse after achieving CRc in patients who received or did not receive prior TKI therapy before treatment with gilteritinib in both trials is shown in Figure S1.

In both trials, responses with gilteritinib therapy were observed in prior TKI-treated patients with baseline *FLT3*-ITD or *FLT3*-TKD mutations as well as in patients harboring both mutation types (Table 3). There was no observed trend in treatment response by *FLT3* mutation type across prior TKI or no prior TKI subgroups. Combined response outcomes from both trials in patients treated with 120-mg gilteritinib show similarly high proportions of patients achieving CRc in both prior TKI therapy (52%) and no prior TKI (53%) groups (Table 4).

**Table 3:** Response outcomes in patients with R/R AML by prior TKI status according to *FLT3* mutation type in the CHRYSALIS and ADMIRAL studies.

CHRYSALIS Trial: 120- or 200-mg Gilteritinib							
Response parameter, *n* (%)	Prior TKI				No Prior TKI		
	*FLT3*-ITD (*n* = 29)		*FLT3*-ITD and -TKD (*n* = 4)		*FLT3*-ITD (*n* = 94)	*FLT3*-TKD (*n* = 9)	*FLT3*-ITD and -TKD (*n* = 7)
CR	1 (3)		0		16 (17)	0	0
CRi	8 (28)		2 (50)		20 (21)	1 (11)	4 (57)
CRp	3 (10)		0		7 (7)	0	0
PR	2 (7)		0		6 (6)	1 (11)	1 (14)
NR	13 (45)		2 (50)		34 (36)	7 (78)	1 (14)
NE	2 (7)		0		11 (12)	0	1 (14)
CRc^a^	12 (41)		2 (50)		43 (46)	1 (11)	4 (57)
**ADMIRAL Trial: 120-mg Gilteritinib**							
**Response parameter,** ***n*** **(%)**	**Prior TKI**				**No Prior TKI**		
	***FLT3*-ITD (*n*** = **24)**	***FLT3*-TKD (*n*** = **5)**		***FLT3*-ITD and -TKD (*n*** = **4)**	***FLT3*-ITD (*n*** = **191)**	***FLT3*-TKD (*n*** = **16)**	***FLT3*-ITD and -TKD (*n*** = **3)**
CR	2 (8)	2 (40)		2 (50)	42 (22)	2 (13)	0
CRi	6 (25)	0		1 (25)	53 (28)	2 (13)	1 (33)
CRp	3 (13)	1 (20)		0	12 (6)	2 (13)	1 (33)
PR	5 (21)	0		0	23 (12)	5 (31)	0
NR	7 (29)	1 (20)		1 (25)	50 (26)	5 (31)	1 (33)
NE	1 (4)	1 (20)		0	11 (6)	0	0
**CRc**^**a**^	**11 (46)**	**3 (60)**		**3 (75)**	**107 (56)**	**6 (38)**	**2 (67)**

Bold font indicates aggregate responses.

^a^Defined as the sum of patients who achieved CR, CRi, and CRc.

*AML* acute myeloid leukemia, *CR* complete remission, *CRc* composite complete remission, *CRi* complete remission with incomplete hematologic recovery, *CRp* complete remission with incomplete platelet recovery, *ITD* internal tandem duplication, *NE* not evaluable, *NR* no response, *PR* partial remission, *R/R* relapsed or refractory, *TKD* tyrosine kinase domain, *TKI* tyrosine kinase inhibitor.

**Table 4:** Combined response outcomes in gilteritinib-treated R/R AML patients by prior TKI status from the CHRYSALIS and ADMIRAL trials.

Response parameter, *n* (%)	120-mg Gilteritinib (*N* = 303)	
	Prior TKI (*n* = 48)	No Prior TKI (*n* = 255)
CR	7 (15)	52 (20)
CRi	13 (27)	67 (26)
CRp	5 (10)	16 (6)
PR	6 (13)	31 (12)
NR	14 (29)	75 (29)
NE	3 (6)	14 (5)
**CRc**^**a**^	**25 (52)**	**135 (53)**

Bold font indicates aggregate responses.

^a^Defined as the sum of patients who achieved CR, CRi, and CRc.

AML acute myeloid leukemia, *CR* complete remission, *CRc* composite complete remission, *CRi* complete remission with incomplete hematologic recovery, *CRp* complete remission with incomplete platelet recovery, *NE* not evaluable, *NR* no response, *PR* partial remission, *R/R* relapsed or refractory, *TKI* tyrosine kinase inhibitor.

### Transplantation and posttransplant gilteritinib maintenance therapy

In the CHRYSALIS trial, 30 patients who received 120- or 200-mg gilteritinib underwent hematopoietic stem cell transplantation (HSCT) and 12 received posttransplant gilteritinib maintenance therapy for a median of 564 days (range, 15–959). Of the 28 patients who underwent transplantation after CRc, 12 (43%) resumed gilteritinib after HSCT. Six patients with prior sorafenib exposure underwent HSCT, all of whom achieved CRc before HSCT (all CRi), but none received posttransplant gilteritinib maintenance therapy. Among patients in the gilteritinib arm of the ADMIRAL trial who received prior TKI therapy, five underwent HSCT during the trial, and four of these five patients resumed gilteritinib after HSCT. Durations of posttransplant gilteritinib therapy for patients with prior midostaurin exposure (*n* = 2) were 1 and 95 days, and for patients with prior sorafenib exposure (*n* = 2) the durations of posttransplant gilteritinib maintenance therapy were 33 and 135 days. Two of the four prior TKI-treated patients who received posttransplant gilteritinib had achieved pretransplant CRc (CRi, *n* = 2 [prior midostaurin, *n* = 1; prior sorafenib, *n* = 1]). Among ADMIRAL patients who had not received prior TKI, 59 underwent HSCT. Of the 38 gilteritinib-arm patients without prior TKI exposure who were transplanted after CRc, 24 (63%) received posttransplant gilteritinib maintenance therapy for a median of 643.5 days (range, 2–1505).

## Discussion

The multi-kinase oral FLT3 TKIs, sorafenib, and midostaurin, are both efficacious in the frontline setting when used in combination with chemotherapy [16, 17, 31] and sorafenib is also beneficial in the post-transplant setting [19, 20] in patients with newly diagnosed *FLT3*-mutated AML. Although not approved for AML, sorafenib was one of the first and most widely used multi-kinase FLT3 TKIs [1]. Midostaurin is a multi-kinase FLT3 TKI approved in combination with high-intensity chemotherapy for patients with newly diagnosed *FLT3*-mutated AML [18]. The availability of more selective *FLT3*-targeted TKI therapies has further expanded treatment options with the approval of gilteritinib for patients with R/R *FLT3*-mutated AML [28]. This is an important clinical advance because outcomes in R/R AML with SC regimens have generally been poor [32]. Because FLT3 TKIs are increasingly being used as frontline therapy for *FLT3*-mutated AML, it is important to understand the degree to which prior TKI therapy alters the likelihood of response or survival benefit conferred by gilteritinib in R/R *FLT3*-mutated AML. Understanding the impact of prior FLT3 TKI therapy on the ability to respond to a subsequent FLT3 TKI might help guide treatment selection in the R/R AML setting.

This analysis of patients from two trials of gilteritinib in the *FLT3*-mutated R/R AML setting demonstrated that a high proportion of patients who received prior midostaurin or sorafenib still achieved remission with gilteritinib. High CRc rates with 120- or 200-mg gilteritinib were observed in heavily pre-treated R/R AML patients in the CHRYSALIS trial (42%) and in R/R AML patients who received 120-mg gilteritinib after a single line of prior induction therapy in the ADMIRAL trial (52%). In both trials, protocol-defined remissions were achieved across patients with *FLT3*-ITD, *FLT3*-TKD, or both mutations. Patients in the gilteritinib arm of the ADMIRAL trial who received prior TKIs had higher response rates than corresponding patients in the SC arm. High response rates with gilteritinib after prior TKI therapy were observed in both relapsed and refractory subgroups; remission duration was shorter in patients who received prior TKIs compared with those who did not. In both the CHRYSALIS and ADMIRAL trials, patients who received prior TKIs before treatment with gilteritinib had shorter remission duration and higher relapse rate compared with patients who did not. Although variability in remission quality from prior therapy likely underlies these differences in remission duration, other factors could have contributed to this observation. For example, per protocol in the CHRYSALIS trial, remission duration was censored prior to HSCT in all patients who proceeded to transplant after CRc. This affected 47% (*n* = 28/60) of CRc responses from the CHRYSALIS trial included in this analysis. Remission durations were also censored prior to transplant for patients in ADMIRAL who did not resume gilteritinib as posttransplant maintenance therapy, although this was relatively uncommon. Nonetheless, censoring of pretransplant remission duration affected a sizeable fraction of patients with prior TKI exposure who achieved CRc in either study (26%; *n* = 8/31). Overall, gilteritinib maintenance therapy was associated with longer survival during ADMIRAL and was administered to the majority of patients in this trial without prior TKI exposure who underwent HSCT after CRc. However, the proportion of patients in ADMIRAL with prior TKI who underwent HSCT after CRc and received gilteritinib maintenance was small, which also potentially contributed to observed differences in remission duration.

In an evaluation of the impact of sequential FLT3 TKI therapy in patients with *FLT3*-mutated AML, Yilmaz and colleagues reported that the rate of CRc declined from 77% for FLT3 TKI therapy in the frontline setting to 31% for TKI therapy administered in the R/R AML setting; the CRc rate further declined to 25% after the third TKI [33]. In a second cohort of patients who received their first TKI in the R/R AML setting, the rate of CRc was 45% after the first TKI and declined to 21% after the second TKI and 12% after the third TKI [33]. Although variations in patient and treatment characteristics render comparison of findings from our analysis to those reported by Yilmaz et al. [33] challenging, it is notable that a considerable proportion (>40%) of patients in our analysis still responded to single-agent gilteritinib after prior TKI therapy. Our observations of shortened OS in prior TKI-treated patients who received gilteritinib in the CHRYSALIS trial concurred with findings reported by Yilmaz and colleagues for patients receiving a second or third FLT3 TKI [33]. However, among patients in the gilteritinib arm of the ADMIRAL trial, median OS was similar among patients who received or did not receive prior TKIs and remained longer than the median OS for corresponding patients in the SC arm. The difference in survival trends related to prior TKI therapy between the CHRYSALIS and ADMIRAL trials may stem from the fact that most patients in the CHRYSALIS trial represented a heavily pretreated population, whereas patients in the ADMIRAL trial had received only one prior line of AML therapy.

Mutations associated with treatment resistance vary between type I and type II FLT3 TKIs [34]. Acquired off-target mutations in *RAS*/*MAPK* pathway genes are most commonly associated with treatment resistance to type I FLT3 inhibitors such as midostaurin and gilteritinib [12, 34]. Acquired *FLT3*-TKD mutations at codon D835 are the most common resistance mutations identified in patients treated with type II FLT3 TKIs sorafenib or quizartinib [6, 34]. In the current analysis, a greater proportion of prior-TKI–treated patients had previously received sorafenib (70%; *n* = 58/83) than midostaurin (30%; *n* = 25/83). As gilteritinib is effective against *FLT3* D835 mutations, the potential acquisition of these mutations likely did not have a negative impact on treatment response. In the current analysis, we observed a CRc rate of 57% (CR, 21%; CRi, 29%; CRp, 7%), among patients who received prior midostaurin before gilteritinib in the ADMIRAL trial. As the use of midostaurin in the frontline setting becomes more prevalent, further investigation of response to gilteritinib as a second FLT3 TKI in the relapsed setting is warranted.

As is commonly seen in secondary analyses, our study was not sufficiently powered to detect significant differences between prior TKI and no prior TKI subgroups, and no adjustments for multiple comparisons were made. Because the numbers of patients who received prior TKI therapy in both trials was small, the results of this study should be interpreted with caution. Variability in prior treatment characteristics and the number of prior lines of AML therapy in the CHRYSALIS and ADMIRAL trials may have also had an impact on the observed outcomes. Furthermore, the presence of a high *FLT3*-ITD allelic ratio at baseline and persistence of measurable residual disease after gilteritinib therapy may have also had an impact on response duration and OS. We did not assess the impact of *FLT3*-ITD allelic ratio in gilteritinib-arm patients previously exposed or not exposed to a FLT3 TKI due to the small number of patients in the ADMIRAL trial with prior FLT3 TKI exposure (*n* = 33) and lack of available samples from the CHRYSALIS study. In addition, we did not evaluate the impact of baseline *RAS/MAPK* pathway mutations on response and survival outcomes in the gilteritinib arm because the small sample size precluded meaningful statistical comparisons between prior TKI–exposed (*n* = 5) and non-exposed (*n* = 13) patients in this co-mutation subgroup. However, some insights can be gleaned from a recent retrospective study of gilteritinib in patients with *FLT3*-mutated R/R AML previously treated with a FLT3 TKI (*n* = 113). Patients harboring *RAS/MAPK* pathway mutations (*n* = 19) had a lower rate of CRc (38%) and shorter median OS (4.9 months) than patients without these mutations (*n* = 62) (CRc = 59%; median OS: 7.8 months, HR = 2.4; 95% CI: 1.1, 5.4; *P* < 0.01) [35].

Findings from this analysis show that patients with *FLT3*-mutated R/R AML who received prior treatment with sorafenib or midostaurin do achieve high remission rates with single-agent gilteritinib. As the use of FLT3 TKIs such as midostaurin becomes more prevalent in the frontline setting, physicians may still consider using gilteritinib as a subsequent FLT3-targeted therapy in the R/R AML setting. Further studies in a larger patient population will help validate these findings and determine the molecular profile(s) of patients for whom gilteritinib as a second FLT3 TKI therapy at relapse fails to improve outcomes relative to alternate regimens.

## Supplementary information

Supplemental Material

## Data Availability

Researchers may request access to anonymized participant level data, trial level data and protocols from Astellas sponsored clinical trials at www.clinicalstudydatarequest.com. For the Astellas criteria on data sharing see: https://clinicalstudydatarequest.com/Study-Sponsors/Study-Sponsors-Astellas.aspx
