# Supplementary material for: Clinical outcomes in patients with relapsed/refractory FLT3-mutated acute myeloid leukemia treated with gilteritinib who received prior midostaurin or sorafenib
Source: Blood Cancer J. 2022 May 30;12(5):84. doi: 10.1038/s41408-022-00677-7 (PMC9151663; doi:10.1038/s41408-022-00677-7)
Supplement: Supplementary file 1 — Supplemental Material [file 41408_2022_677_MOESM1_ESM.docx]

**SUPPLEMENTARY INFORMATION**

**Table S1. Definitions of Response Parameters**

| **Response Parameter** | **Definition** |
| --- | --- |
| **Complete remission (CR)** | Presence of regenerating hematopoietic cells in the bone marrow and achievement of a morphologic leukemia-free state with absolute neutrophil count (ANC) ≥1 × 10^9^/L, platelet count ≥100 × 10^9^/L, normal bone marrow differential with <5% blasts, and red blood cell (RBC)/platelet transfusion independence with no evidence of extramedullary leukemia. |
| **Complete remission with partial hematologic recovery (CRh)** | Bone marrow blasts <5% with partial hematologic recovery defined as ANC ≥0.5 × 10^9^/L and platelet count ≥50 × 10^9^/L, with no evidence of extramedullary leukemia and cannot be classified as CR. |
| **Complete remission with incomplete platelet recovery (CRp)** | Achievement of all CR criteria except for platelet recovery (platelet count <100 × 10^9^/L). |
| **Complete remission with incomplete hematologic recovery (CRi)** | Achievement of all CR criteria except for hematologic recovery with residual neutropenia (ANC <1 × 10^9^/L) with or without RBC/platelet transfusion independence. |
| **Composite complete remission (CRc)** | Achievement of CR, CRi, or CRp. |
| **Partial remission (PR)** | Presence of regenerating normal hematopoietic cells in bone marrow with evidence of peripheral recovery with no (or only a few regenerating) circulating blasts and with a ≥50% decrease in the number of blasts in the bone marrow aspirate with total marrow blasts between 5% and 25%. A value of ≤5% blasts is also considered a PR if Auer rods are present. |
| **Relapse** | - Relapse after CR, CRh, CRp, or CRi:   - Reappearance of leukemic blasts in the peripheral blood and a ≥5% increase in the percentage of blasts that is not attributable to any other cause, or reappearance or new appearance of extramedullary leukemia - Relapse after PR:   - Reappearance of significant numbers of peripheral blasts and an increase in the percentage of blasts in the bone marrow aspirate to >25% not attributable to any other cause, or reappearance or new appearance of extramedullary leukemia |
| **Overall survival (OS)** | Time from the date of randomization until the date of death from any cause. For subjects who are not known to have died by the end of study follow-up, OS was censored at the date of last contact. |
| **Event-free survival (EFS)** | Time from the date of randomization until the date of documented relapse (excluding relapse after PR), treatment failure, or death, whichever occurs first. For subjects who are not known to have had a relapse, treatment failure, or death event, EFS was censored at the date of last relapse-free disease assessment. |

**Table S2. Median Overall Survival by Prior TKI Status Based on *FLT3* Mutation Type in Gilteritinib-Treated Patients With R/R AML: CHRYSALIS and ADMIRAL Trials**

| ***FLT3* Mutation Type** | **CHRYSALIS**  **120- or 200-mg Gilteritinib** | | **ADMIRAL**  **120-mg Gilteritinib** | |
| --- | --- | --- | --- | --- |
|  | **Median OS, months (95% CI)** | | **Median OS, months (95% CI)** | |
|  | **Prior TKI** | **No Prior TKI** | **Prior TKI** | **No Prior TKI** |
| *FLT3*-ITD only | 7.5 (4.1, 9.4) | 7.5 (5.0, 10.8) | 8.7 (4.5, 10.8) | 9.6 (7.2, 11.0) |
| *FLT3*-TKD only | NA | 4.3 (1.8, 8.1) | 4.6 (1.2, 24.1) | 8.0 (3.0, 24.6) |
| *FLT3*-ITD and -TKD | 5.6 (1.9, 8.7) | 9.6 (1.4, 20.4) | 13.2 (4.0, NE) | 10.2 (8.9, 20.2) |

Abbreviations: AML, acute myeloid leukemia; CI, confidence interval; ITD, internal tandem duplication; NA, not applicable; NE, not estimable; OS, overall survival; R/R, relapsed or refractory; TKD, tyrosine kinase domain; TKI, tyrosine kinase inhibitor.

**Table S3. Median Overall Survival and Response Outcomes by Prior TKI Status in the Gilteritinib Arm of the ADMIRAL Trial: Relapsed vs Refractory Status**

| **Parameter** | **Relapsed** | | **Refractory** | |
| --- | --- | --- | --- | --- |
|  | **Prior TKI**  **(n=19)** | **No Prior TKI**  **(n=130)** | **Prior TKI**  **(n=14)** | **No prior TKI**  **(n=84)** |
| **Median OS, months (95% CI)** | 6.5  (4.0, 11.3) | 8.9  (6.7, 10.8) | 10.6  (3.5, 24.1) | 10.2  (7.9, 13.3) |
| **Response, n (%)** | | | | |
| CR | 3 (16) | 32 (25) | 3 (21) | 14 (17) |
| CRi | 4 (21) | 37 (39) | 3 (21) | 19 (23) |
| CRp | 2 (11) | 9 (7) | 2 (14) | 6 (7) |
| PR | 5 (26) | 12 (9) | 0 | 16 (19) |
| NR | 4 (21) | 34 (26) | 5 (36) | 23 (27) |
| NE | 1 (5) | 6 (5) | 1 (7) | 6 (7) |
| **CRc^a^** | **9 (47)** | **78 (60)** | **5 (57)** | **39 (46)** |

Bold font indicates aggregate responses.

^a^Defined as the sum of patients who achieved CR, CRi, and CRp.

Abbreviations: CI, confidence interval; CR, complete remission; CRc, composite complete remission; CRi, complete remission with incomplete hematologic recovery; CRp, complete remission with incomplete platelet recovery; NE, not evaluable; NR, no response; OS, overall survival; PR, partial remission; TKI, tyrosine kinase inhibitor.

**Figure S1. Cumulative Incidence of Relapse by Prior TKI Status in Patients With R/R AML Who Received Gilteritinib**


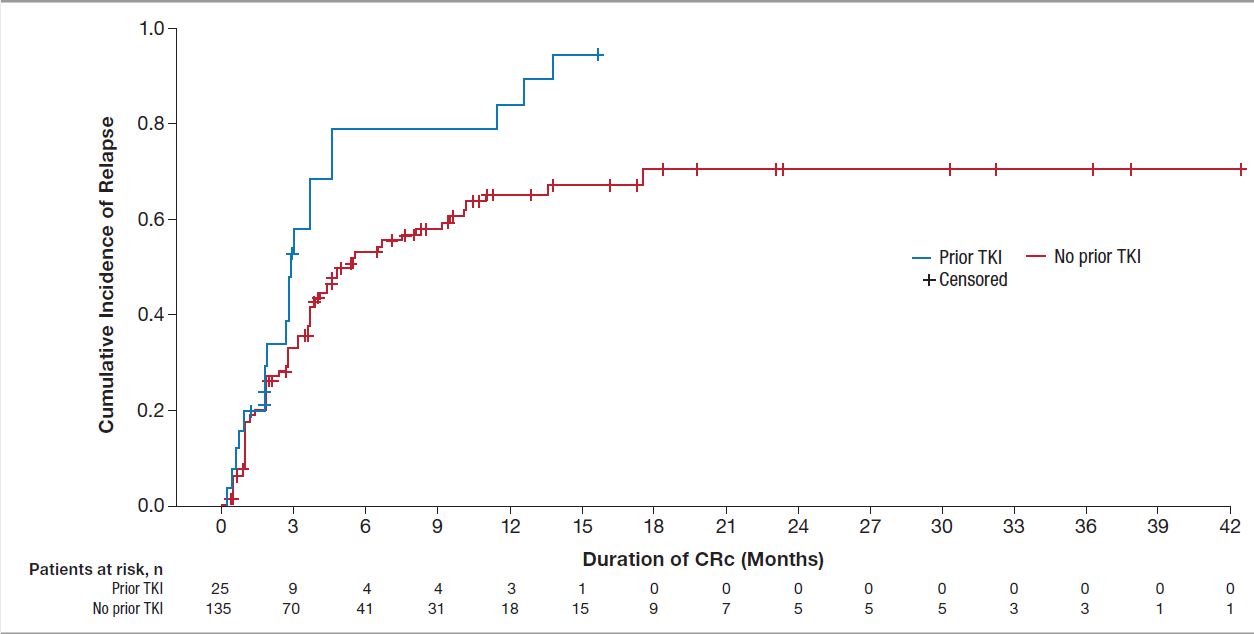


Abbreviations: AML, acute myeloid leukemia; CRc, composite complete remission; R/R, relapsed or refractory; TKI, tyrosine kinase inhibitor.
